# A new framework for warehouse assessment using a Genetic-Algorithm driven analytic network process

**DOI:** 10.1371/journal.pone.0256999

**Published:** 2021-09-07

**Authors:** Wafa’ H. AlAlaween, Abdallah H. AlAlawin, Mahdi Mahfouf, Omar H. Abdallah, ‎Mohammad A. Shbool, Mahmoud F. Mustafa

**Affiliations:** 1 Department of Industrial Engineering, The University of Jordan, Amman, Jordan; 2 Department of Industrial Engineering, The Hashemite University, Zarqa, Jordan; 3 Department of Automatic Control and Systems Engineering, The University of Sheffield, ‎Sheffield, England, United Kingdom; 4 Dnata, Queen Alia Airport, Amman, Jordan; 5 The Conformity Assessment Centre, The Royal Scientific Society, Amman, Jordan; Gonbad Kavous University, ISLAMIC REPUBLIC OF IRAN

## Abstract

A novel way of integrating the genetic algorithm (GA) and the analytic network process (ANP) is presented in this paper in order to develop a new warehouse assessment scheme, which is developed through various stages. First, we define the main criteria that influence a warehouse performance. The proposed algorithm that integrates the GA with the ANP is then utilized to determine the relative importance values of the defined criteria and sub-criteria by considering the interrelationships among them, and assign strength values for such interrelationships. Such an algorithm is also employed to linguistically present the relative importance and the strength of the interrelationships in a way that can circumvent the use of pairwise comparisons. Finally, the audit checklist that consists of questions related to the criteria is integrated with the proposed algorithm for the development of the warehouse assessment scheme. Validated on 45 warehouses, the proposed scheme has been shown to be able to identify the warehouse competitive advantages and the areas where more improvements can be achieved.

## 1. Introduction

Market globalization and the ability of reaching-out to a wider scope of customers should enable enterprises to increase their sales and profit, and to gain leverage in such a highly competitive environment [1]. However, the rapid spike in sales has become overwhelming in today’s highly competitive environment that, more often than not, is characterised by high levels of uncertainties. Consequently, supply chain and logistics have been receiving more and more interest. Nowadays, enterprises outsource their supply chain activities to third-party logistics providers (3PLPs) in order to focus on their core businesses, improve service quality and corporate image and minimize cost. Because of the number of 3PLPs available, evaluating them is an indispensable process that proceeds eliciting the best one. Such a task, as a multi-criteria decision-making (MCDM) case, is not as simple as it may seem at first glance, this being due to the conflicting multi-criteria that need to be taken into account [2].

A considerable number of research papers and books has hitherto been devoted to addressing the task of selecting the best 3PLP [2, 3]. Such studies have focused on (i) identifying the related criteria and sub-criteria such as delivery time and quality; and (ii) investigating different MCDM algorithms that can be employed to assess 3PLPs. The MCDM approaches that have hitherto been presented in the literature have, in general, their own strengths and limitations. For instance, the analytic hierarchy process (AHP) approach can take into consideration human judgment (e.g. subjective information). However, it cannot take into account the interrelationships among defined criteria or constraints [4]. Therefore, various algorithms that integrate two or more of these approaches have also been developed [5, 6]. Most of the research papers and books have evaluated warehouses and their logistics as a part of the whole 3PLP assessment, in other words such research work has only performed the assessment as a holistic view of 3PLPs without considering the detail levels. Since a warehouse and its logistics account for approximately 70% of the total incurred cost and, thus, can considerably influence the warehouse performance [7], assessing warehouses and their logistics can provide mangers with a better idea about these and can allow enterprises to select the optimal solution more reliably. Therefore, a comprehensive assessment scheme for warehouses based on the novel integration between the genetic algorithm (GA) and the analytic network process (ANP) is proposed. The motivation behind such an integration stems from the need to (i) determine stable relative importance values of the criteria in a way that considers the interrelationships among them, (ii) numerically represent the strength values of the interrelationships among the criteria and their corresponding sub-criteria, and (iii) linguistically represent the importance of the criteria and the strength of the interrelationships in a way that can circumvent, in the future, the difficulty of collecting the relative importance of the criteria using a considerable number of pairwise comparisons. The development of such a scheme starts by identifying the criteria that influence the warehouse performance. The GA, as an evolutionary computing algorithm, is integrated with the ANP approach. Such an integration is, finally, employed to assess warehouses and their logistics based on the defined criteria and sub-criteria that are represented in a checklist form that consists of all related questions. This paper is organised as follows: the related research work is summarized in Section 2. The criteria are then presented in Section 3, whereas the proposed algorithm is presented in Section 4. The new assessment scheme, its associated algorithm, and the results obtained are outlined in Section 5, whereas conclusions and future research directions are given and discussed in Section 6.

## 2. Literature review

A considerable number of research papers and books has hitherto been devoted to addressing the task of selecting the best 3PLP [2, 3]. Some researchers have even focused on identifying the related criteria and sub-criteria (e.g. delivery time, quality, cost, expertise, service innovation, information system and flexibility) [8], whereas other researchers have studied different MCDM algorithms that can be employed to assess 3PLPs [9]. Qualitative as well as quantitative approaches have been utilized in the related literature [10]. For example, a simple assessment form was proposed in order to allow manager to obtain more information about the distribution facilities from tour visits [11]. Furthermore, cost efficiency and cost effectiveness analysis were utilized to assess warehouse processes based on different criteria that include, but not limited to, flow, storage space, routes travelled by employees, load time and stability of employment [12]. In addition, the AHP was employed to assess the performance of 3PLPs when social sustainability was considered [13]. Furthermore, the ANP has been also utilized to compensate for the assumption that the criteria are independent which is usually assumed when the AHP approach is implemented [4]. For instance, the ANP approach was utilized to classify different 3PLPs into three levels categories based on various criteria (e.g. cost, compatibility, operational performance and risk management) [14]. The linear optimization algorithms have also been developed to evaluate 3PLPs. For example, various contracts presented by 3PLPs were investigated using a linear optimization paradigm [15]. Data Envelopment Analysis (DEA) was also used to determine the efficiency [16]. Recently, artificial intelligence models have been included in many applications, such as manufacturing and marine, this being due to their ability to mimic the human cognitive process [17, 18]. Such models have also been implemented in the area of logistics and supply chain management to incorporate human expertise. For instance, interval type-2 fuzzy sets were integrated with the inter-criteria correlation and weighted sum-product assessment to evaluate different 3PLPs in an uncertain environment [7]. Likewise, a new fuzzy pivot pairwise relative criteria importance assessment approach was proposed to assess the environment for the implementation of barcode technology in a warehouse system based on 27 criterion [19]. In addition, an approach based on fuzzy logic was presented to evaluate various 3PLPs by focusing on their warehouses and their related activities [3].

The MCDM approaches that have hitherto been presented in the literature to evaluate 3PLPs have, in general, their own strengths and limitations. For instance, the AHP approach can take into consideration human judgment (e.g. subjective information). However, it cannot take into account the interrelationships among defined criteria or constraints [4]. Therefore, various algorithms that integrate two or more of these approaches have also been developed, this being due to the fact that the integration of two or more algorithms allows users to circumvent the limitations of applying one algorithm. An algorithm that integrated a neural network with fuzzy logic was employed in the reverse logistics [5]. In addition, the real time Delphi was integrated with the ANP approach in order to assess a warehouse performance using 42 criterion [20]. A fuzzy AHP based on the distance from the average solution was proposed to assess 3PLPs [6]. Likewise, case- and rule-based reasoning were integrated with fuzzy compromise programming paradigms in order to effectively evaluate 3PLPs. In this research work, a novel MCDM algorithm based on the integration between the GA and the ANP is proposed.

## 3. Criteria

Various criteria that influence a warehouse performance and its logistics activities were identified, in this research work, by a thorough review of the literature and consulting experts in the area of warehouse science and supply chain management via an online survey and through structured meetings. It is worth mentioning at this stage that approximately 120 responses were received. In addition to the main criteria, the corresponding sub-criteria and their sub-sub-criteria were also identified. The main ten (10) criteria and their sub-criteria/sub-sub-criteria are briefly described in the following sub-sections.

### 3.1 Facilities

In addition to its significant effect on local and global supply chain networks, facilities can significantly affect the logistics activities. To illustrate, a warehouse location in a local or global supply chain network can affect, for example, delivery lead-time, customer support, cost and market expansion [21]. The five sub-criteria and their corresponding sub-sub-criteria are listed in Table 1.

**Table 1:** The defined sub-criteria and the coreesponding sub-sub-criteria for the facilities.

Sub-criteria	Sub-sub-criteria
Location	• Transport network,
	• Particular requirements(e.g. energy and water utilities),
	• Longevity of a location,
	• Financial issues (e.g. taxes),
	• Policies and regulations.
Number of locations	• Optimal number of locations,
	• Inbound and outbound transportation,
	• Protocol for urgent orders.
Layout	• Workstations and activities space,
	• Floor surface requirements (e.g. maximum load),
	• Support area,
	• Efficient flow,
	• Ergonomic layout requirements,
	• Design flexibility.
Work conditions and workplace environment	• Temperature and humidity control,
	• Efficient illumination system,
	• Efficient ventilation system,
	• Monitoring noise and vibration levels,
	• Key performance indicators for various issues (e.g. work time),
	• Health and safety.
Security	• Securing the area (e.g. surveillance systems),
	• Safety and security training,
	• Rules and regulation for visiting.

### 3.2 Material handling equipment

Material handling equipment is not only a criterion that significantly affects a warehouse performance without directly adding value to its products, but it also affects other criteria such as safety. In general, it accounts for approximately 25% of the manufacturing costs [22]. In addition to this, it is considered to be extremely important because it accounts for 21% of permanent disabilities, and it improves warehouse efficiency and layout [22]. Therefore, it can be evaluated by (i) the use of the best equipment, (ii) periodical tests and preventive maintenance, and (iii) risk assessment as well as safety training and instructions.

### 3.3 Products

Warehouse operations are mostly related to products such as assembling products, delivery activities, shipping orders and many more operations. These activities can include, but not limited to, a labelling system, a product traceability (e.g. radio frequency identification) and a waste management program.

### 3.4 Processes

In general, warehouse processes are almost the same despite the differences in the functions, the products that are dealt with and management [21]. Eleven warehouse processes are defined as presented in [3]. For instance, the receiving process can be assessed by having a receiving protocol, an efficient system to document all the related information, well-known health and safety instructions particularly when the warehouse deals with chemical or heavy items, and a non-conforming items protocol.

### 3.5 Warehouse management system

A warehouse management system (WMS) is usually designed to effectively support warehouse operations and activities by preparing and managing daily plans, and organizing and effectively controlling warehouse resources [23]. The efficient protected WMS is the one that involves all operations and their importance and can be integrated with various systems.

### 3.6 Energy efficiency

Nowadays, the move to a ‘greener’ and energy-efficient warehouse is a target that warehouse and enterprise managers strive to achieve. In general, warehouses can significantly reduce the cost related to energy without any considerable investment [24]. Therefore, energy efficiency needs to be carefully assessed by evaluating the use of an efficient energy system.

### 3.7 Ethics

In today’s industrial environment, codes of ethics and conduct can significantly affect the world economic system by influencing the international trade and transactions, and human rights [25]. In addition to this, the ethical duties towards employees, clients, companies and the nation are considered to be a key for a successful business. Therefore, such codes need to be evaluated in a warehouse by a number of related questions such as “Does the organization have an anti-corruption code of conduct?”

### 3.8 Safety

In general, the number of accidents and injuries that may occur in warehouses is relatively large when compared to other facilities [26]. Therefore, strict rules and regulations related to occupational safety and health are, more often than not, imposed and followed to ensure a safe work environment. As a result, various audit questions related to safety, hazard codes and contingency plan need to be included in the comprehensive assessment scheme.

### 3.9 Quality management system

A quality management system has usually been implemented to improve warehouse processes and logistics activities, even though it has not been well-publicized in the related literature [27]. This system can be evaluated by considering system documentation, internal audit and performance measurements, and preventive and corrective actions.

### 3.10 Human resources system

The success of a warehouse, in general, depends on its human resources and staff knowledge, experience and creativity. Likewise, effective human resources system has several potential advantage for not only a warehouse but also for an enterprise, in particular, a 3PLP [28]. In order to ensure they are effective, human resources need, therefore, to be assessed in terms of, for instance, training and development and resources planning.

## 4. The proposed algorithm

Decision-making is, in general, a basic concept of the cognitive process, where one needs to select the best or a satisfactory alternative among other ones. However, such a process is indeed not simple to implement as it may seem, this being due to the conflicting criteria that need to be considered and the high level of uncertainties that need to be dealt with [2]. Therefore, a MCDM process, as a framework that systematically supports the decision-making process in complex cases, has been extensively employed in many applications (e.g. healthcare and manufacturing) in order to obtain a reliable and reasonable decision [29]. Various algorithms have been presented under the umbrella of the MCDM process. These algorithms include an AHP, an ANP, DEA, case-based reasoning, GA and fuzzy logic [3, 6, 16]. Although these algorithms are different, they are based on the basic idea of evaluating the performance of various courses of actions or alternatives with respect to predefined criteria that are associated with the case under investigation. In general, these algorithms have their own strengths and limitations. Therefore, integrating two or more algorithms, when selected carefully, can combine the strengths of the single algorithms involved in such a way that can circumvent the potential limitations of implementing each algorithm alone. In this research work, an integration of the ANP approach and the GA is, therefore, proposed. The motivation behind such an integration stems from the need to (i) determine stable relative importance values of the criteria in a way that considers the interrelationships among them, (ii) numerically represent the strength values of the interrelationships among the criteria and their corresponding sub-criteria, and (iii) linguistically represent the importance of the criteria and the strength of the interrelationships in a way that can circumvent, in the future, the difficulty of collecting the relative importance of the criteria using a considerable number of pairwise comparisons.

**Fig 1:**
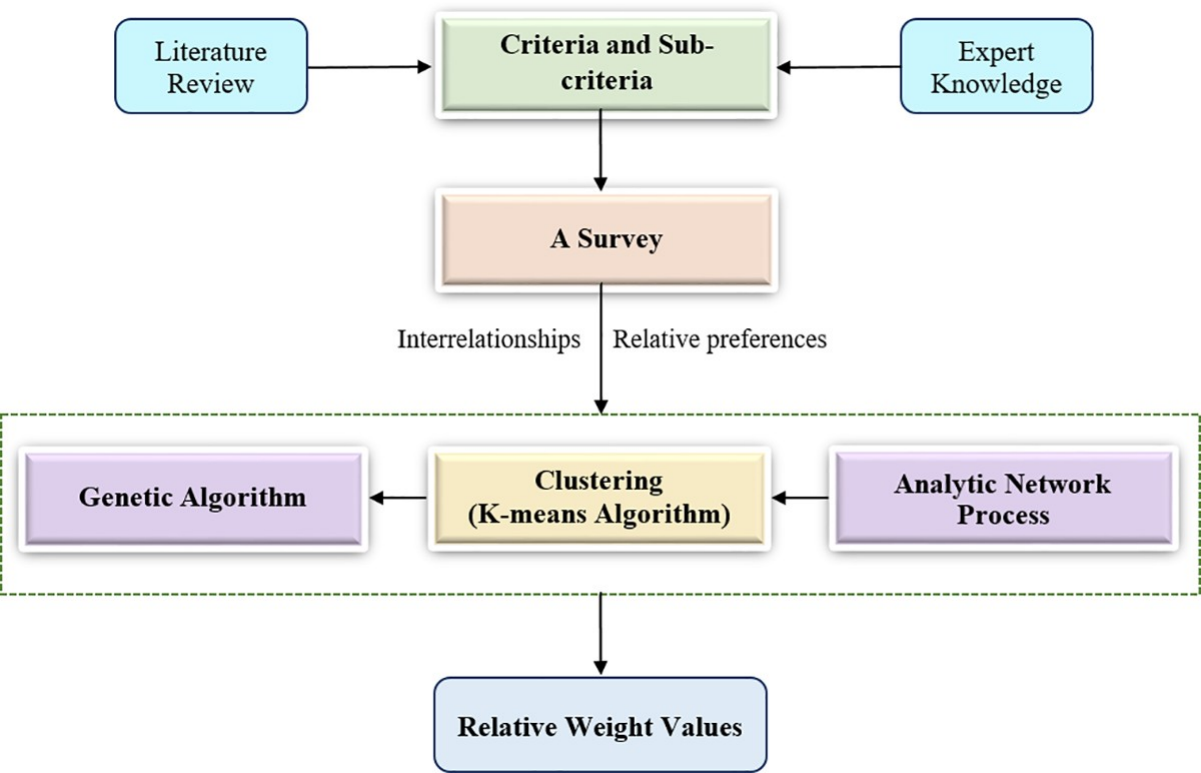
The integrated approach between the GA and the ANP approach.

Fig 1 is a schematic representation of the proposed integration. The defined criteria and sub-criteria that are associated with the case under investigation are identified by a thorough review of the literature and consulting experts in the related area. A survey was conducted to estimate the relative preferences of the criteria and sub-criteria by pairwise comparisons, and to determine the interrelationship among them. Based on the preferences obtained, the ANP approach is then implemented in order to develop the super-matrices and to evaluate the priority vectors. The criteria and sub-criteria are classified into three clusters, namely, significantly important, important and insignificantly important, based on their priority vectors. Here, the parameters of each cluster (i.e. mean and standard deviation) are also estimated. This is followed by determining the membership degree of each criterion and sub-criterion to these three clusters. In order to determine the membership degree, the following equation can be used:

$$\mu_{ij}^{c}=exp\left(\frac{-{\left(x_{i}^{c}-M_{j}^{c}\right)}^{2}}{2\times {\left(\sigma_{j}^{c}\right)}^{2}}\right)$$ (1)

where $\mu_{ij}^{c}$ is the membership degree of the *i*th criterion to the *j*th cluster for product class *c*. The parameter $x_{i}^{c}$ stands for the relative weight of the *i*th criterion for product class c, whereas the parameters $M_{j}^{c}$ and $\sigma_{j}^{c}$ represent the mean and the standard deviation of the *j*th cluster for the product class *c*.

The GA, as a metaheuristic algorithm that is inspired by the natural selection process, is employed to define the optimal value of the membership degrees that correspond to the optimal values of the parameters of the defined clusters. In general, the optimal values can be defined when the GA converges by either reaching an acceptable performance value or reaching a predefined number of iterations. In addition, the strength values of the interrelationships among the main criteria and their sub-criteria are also evaluated. Finally, the relative importance values (i.e. the global priority vector) are estimated for each criterion by the weighted average method of the membership degrees associated with the three clusters.

The theoretical background of the ANP and GA has been well-publicised. For more details, readers can refer to various related books and articles [30, 31].

## 5. The warehouse assessment scheme: Methodology

### 5.1 Implementation and results

**Fig 2:**
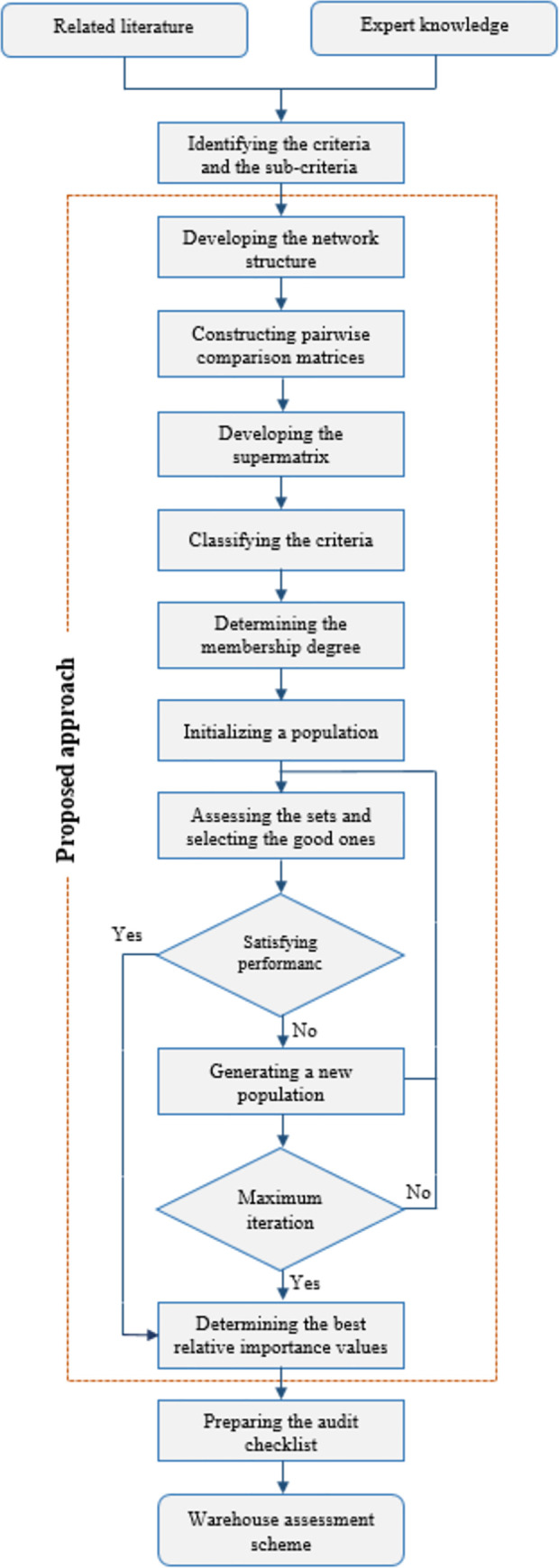
Flow chart of the warehouse assessment scheme development.

The assessment scheme, which is based on the novel integration of the GA and the ANP approach, was developed. The steps that were followed to develop such a scheme are summarized in Fig 2.

#### 5.1.1 Identifying the criteria

The criteria and their corresponding sub-criteria that influence the warehouse performance were identified. Such criteria and sub-criteria were briefly described in the third section. In order to develop a comprehensive and accurate scheme, an audit checklist that contains questions related to these criteria and the corresponding sub-criteria and sub-sub-criteria was prepared. To illustrate, a “Warehouse Processes” criterion has 11 various processes that are considered to be sub-criteria. Each process has sub-sub-criteria. For instance, the sub-sub criteria for the Picking process are related to (i) a defined picking protocol, (ii) key performance indicators (KPIs), and (iii) instructions. Several questions were stated to assess the sub-sub-criteria effectively. For example, the KPIs can be assessed by a number of questions such as “Does the organization have a KPI to measure the accuracy of the picking process?”

#### 5.1.2 Developing the network structure

**Fig 3:**
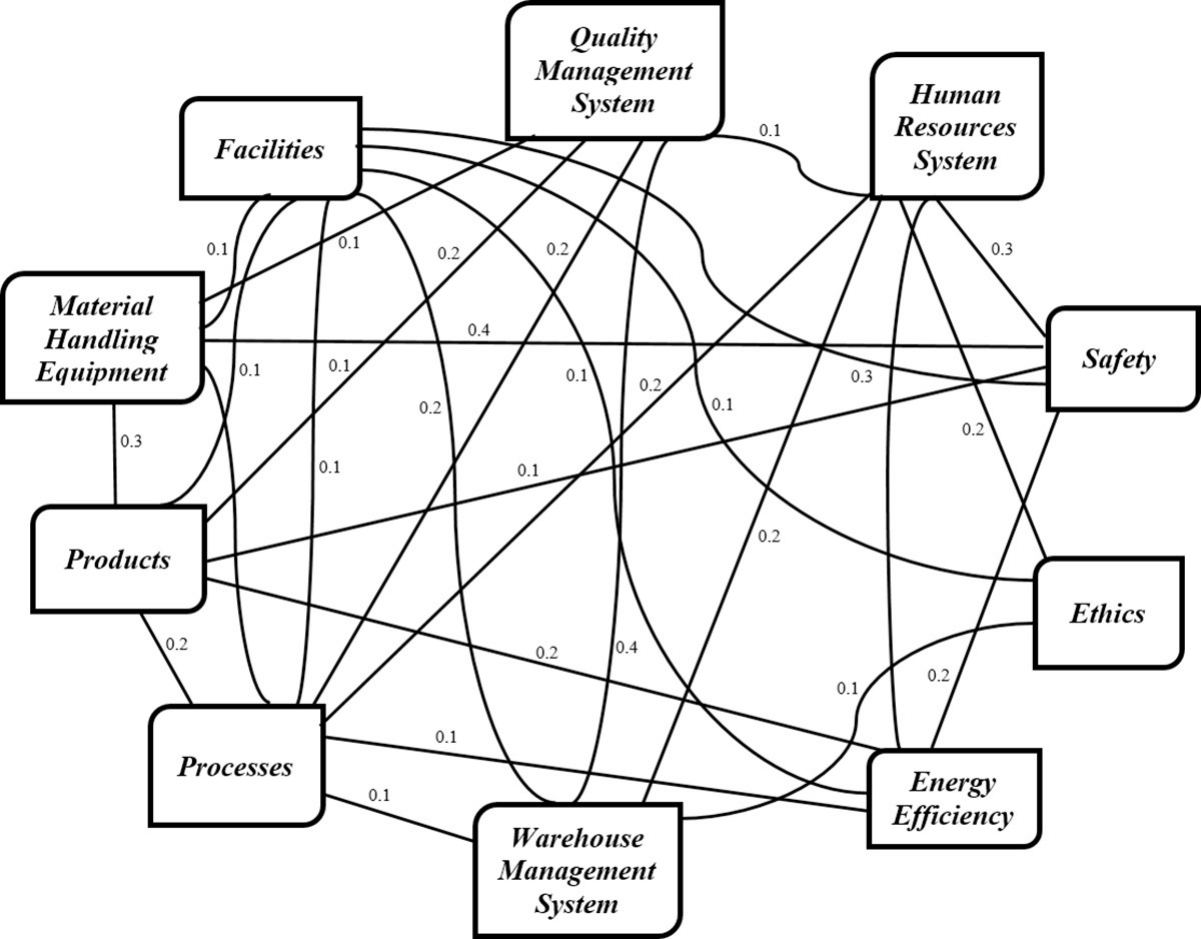
The network structure with the strength values of the interrelationships.

To develop such a structure, one has to identify the interrelationships among the main criteria and sub-criteria which were defined by the related literature and expert knowledge. In this research paper, the interrelationships were defined based on an online survey. It is worth mentioning at this stage that 400 responses were collected for this purpose. The network structure for the 10 criteria is schematically represented in Fig 3. At this stage it is worth emphasising that the interrelationships among the sub-sub-criteria and audit questions were not explicitly considered.

#### 5.1.3 Constructing pairwise comparison matrices

Using the survey that was distributed online, a number of pairwise comparisons was employed to determine the elements (e.g. criteria and sub-criteria) relative importance with other elements. To this effect, a grading system with a scale of 1 to 5 was employed. It is rational that such a relative importance depends significantly on the product(s) that warehouses deal with. For instance, monitoring and control work conditions (e.g. temperature) are extremely important for food warehouses. However, these are not particularly important (i.e. may be considered to be negligible) for metal and manufacturing equipment warehouses. Therefore, the relative importance and the corresponding pairwise comparison matrices should be determined and constructed for the different classes of products. In order to perform this successfully, products need to be classified accordingly. For such a purpose, NICE classification that consists of 34 classes was utilized in this research work [32]. For instance, Class 25 consists of clothing, footwear and headwear. In this research work, various pairwise comparison matrices were developed for the different product classes.

#### 5.1.4 Developing the super-matrix

**Fig 4:**
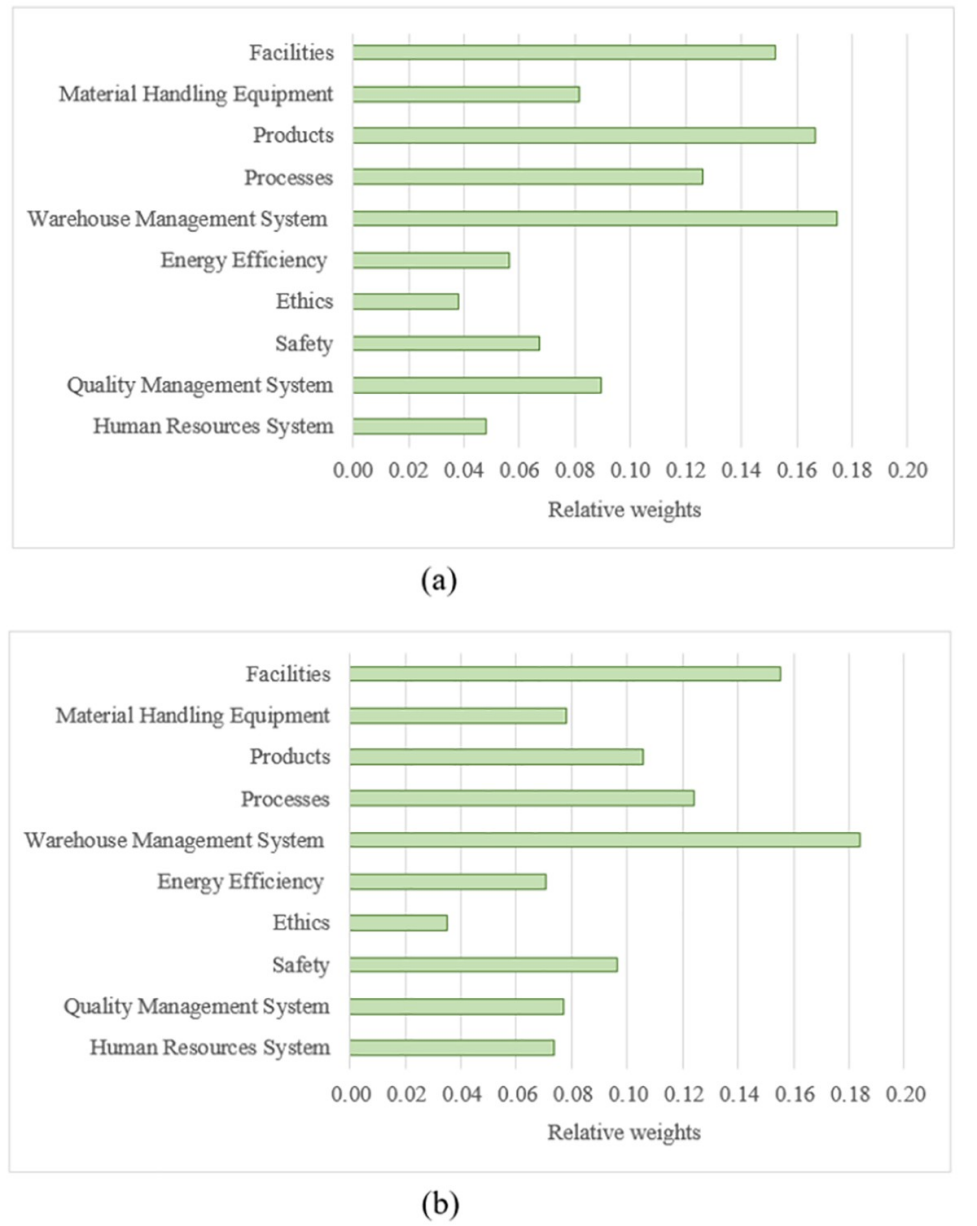
The relative weights of the main criteria for two product classes. (a) Class 5: Pharmaceuticals, medical and veterinary preparations; etc. and (b) Class 25: Paper and cardboard; etc.

Once the pairwise comparison matrices for the criteria and their sub-criteria were collected for the different classes of the products, and the interrelationships were carefully identified, the super-matrices were constructed and the relative weight values were determined. It is worth emphasising that a difference in the relative weight values of the defined criteria for the 34 classes was noticed. For instance, the relative weight values for Classes 5 and 16, can be seen in Fig 4.

As can be seen in the same figure, the relative weight values significantly depend on the product classes. For example, the relative weight values of the “Products” and “Quality Management System” criteria are relatively considerable for Class 5 (i.e. Pharmaceuticals, medical and veterinary preparations; etc.) compared to that for Class 16 (i.e. Paper and cardboard; printed matter; etc.). This can be attributed to the nature of the products and the special conditions required during the various processes (e.g. storing and receiving). Furthermore, the relative weight values of the “Facilities”, “Material Handling Equipment”, “Processes”, “Warehouse Management System” and “Ethics” criteria are relatively the same for the two classes. However, the weight values of the “Energy Efficiency”, “Safety” and “Human Resources System” are relatively high for Class 16 when compared to that for Class 5. These criteria need to have relatively the same importance, regardless of the product classes, as the majority of experts believe this may be the case.

**Fig 5:**
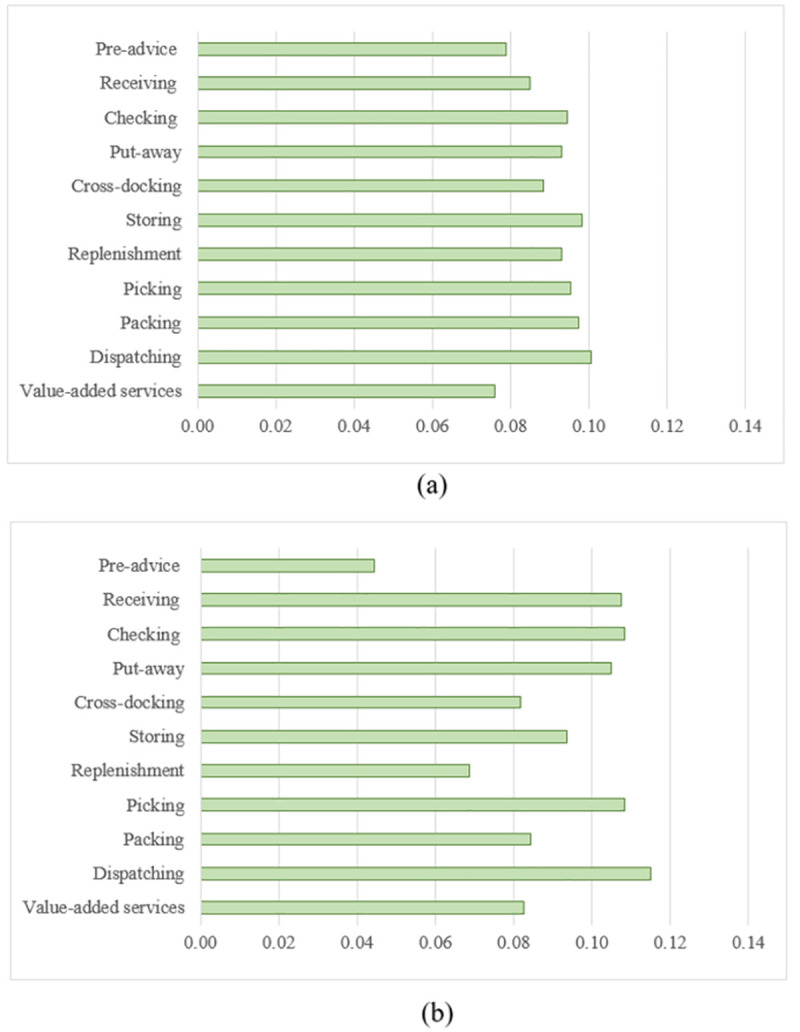
The relative weights of the processes, as sub-criteria, for two product classes. (a) Class 5: Pharmaceuticals, medical and veterinary preparations; etc. and (b) Class 25: Paper and cardboard; etc.

The relative weight values of the various processes, as examples of the sub-criteria, for Classes 5 and 16 are presented in Fig 5. For Class 5, the relative weight values for the 11 processes are in the range of 0.08 to 0.1, as presented in Fig 5(A). Therefore, one can conclude that the relative importance of these processes is almost the same. However, it is apparent that there is a significant difference in the relative importance of these processes when Class 16 is considered, as shown in Fig 5(B). To illustrate, the “Receiving”, “Checking”, “Put-away” and “Dispatching” processes have the maximum values of the relative weights, whereas the “Pre-advice” process has approximately half of the value assigned to these processes, this perhaps being due to the nature of the products included in Class 16 where there is no need for rigorous arrangements.

#### 5.1.5 Classifying the criteria

**Fig 6:**
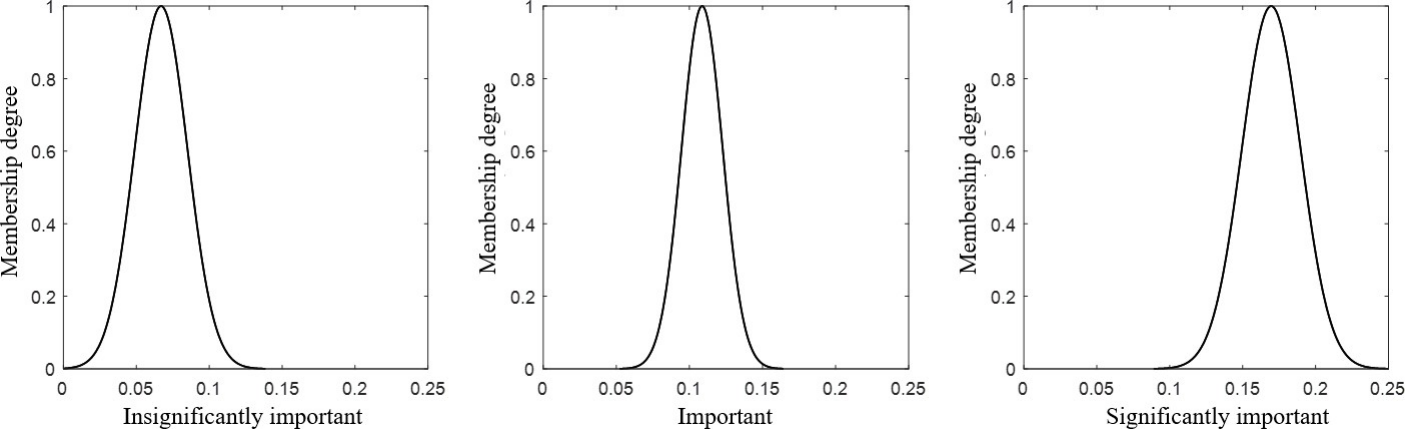
The three clusters of the main criteria for class 16.

For each product class, the criteria were classified into three clusters: significantly important, important and insignificantly important. Such a classification was based on the criteria relative importance values. It is worth mentioning that the K-means clustering approach was employed in this research paper. Fig 6 shows the three clusters for Class 16, as an example. The “Facilities” and “Warehouse Management System” criteria belong to the significantly important cluster, whereas the “Products”, “Processes” and “Safety” criteria belong to the important cluster. The remaining criteria belong to the insignificantly important cluster. In addition, the sub-criteria were also classified into three clusters. Likewise, the interrelationships among the criteria were also classified into the three mentioned clusters.

#### 5.1.6 Determining the membership degree

Once the clusters and their parameters were identified, the membership degrees for the 10 criteria to the three clusters were determined using Eq (1).

#### 5.1.7 Initializing a population

A population that consisted of 300 sets of parameters for the three clusters mentioned previously was randomly initialized. Such parameters were then coded as strings. In this research work, six-bit binary strings were used to define these parameters. Such strings were then concatenated and mapped to decimal values.

#### 5.1.8 Assessing the sets of the parameters and selecting a set of the good ones

The fitness function that was utilized to assess the parameters was the square of the error values that resulted from the estimation of the membership degrees for the criteria, sub-criteria and the strength of the interrelationships. The average of the fitness values was then employed to calculate the relative fitness of each set of parameters. Such values were utilized to elicit the good sets by using a threshold value.

#### 5.1.9 Generating a new population

A new population was generated using the good sets of the parameters via a combination of the three genetic operations, namely, crossover, reproduction and mutation. Such a new population was then evaluated using the fitness function. Such a process was repeated until the number of iterations reached a predefined value.

#### 5.1.10 Determining the best relative importance values

The relative importance values of the criteria and the sub-criteria, and the strength values of the interrelationships among the criteria were finally determined by the weighted average method of the membership degrees associated with the three clusters. The strength values of the interrelationships which were obtained by the GA for Class 16, as an example, are presented in Fig 3. It is worth mentioning at this stage that the strength value of interrelationship between two criteria is assumed to be the same, in other words the effect of first criterion on a second one is equal to the effect of the second criterion on the first one.

#### 5.1.11 Preparing the audit checklist

The audit checklist that contains the corresponding questions was prepared. By such a checklist, the performance of a warehouse can be assessed. For example, “Does the organization have/design a material handling system?” question should be answered by “Conforms”, “Minor non-conformance”, “Major non-conformance” or “Opportunities for improvement”. When the answer is one of the first three answers, the warehouse in the organization should be given the corresponding weight value or fraction of it, whereas a zero value is assigned when the answer is “Opportunities for improvement”. Once all audit questions have been answered, the weight values are added up to determine an overall performance value that will be in the range of 0 to 1. By integrating the checklist and the proposed algorithm, the warehouse assessment scheme was successfully completed.

### 5.2 Warehouses assessment

**Fig 7:**
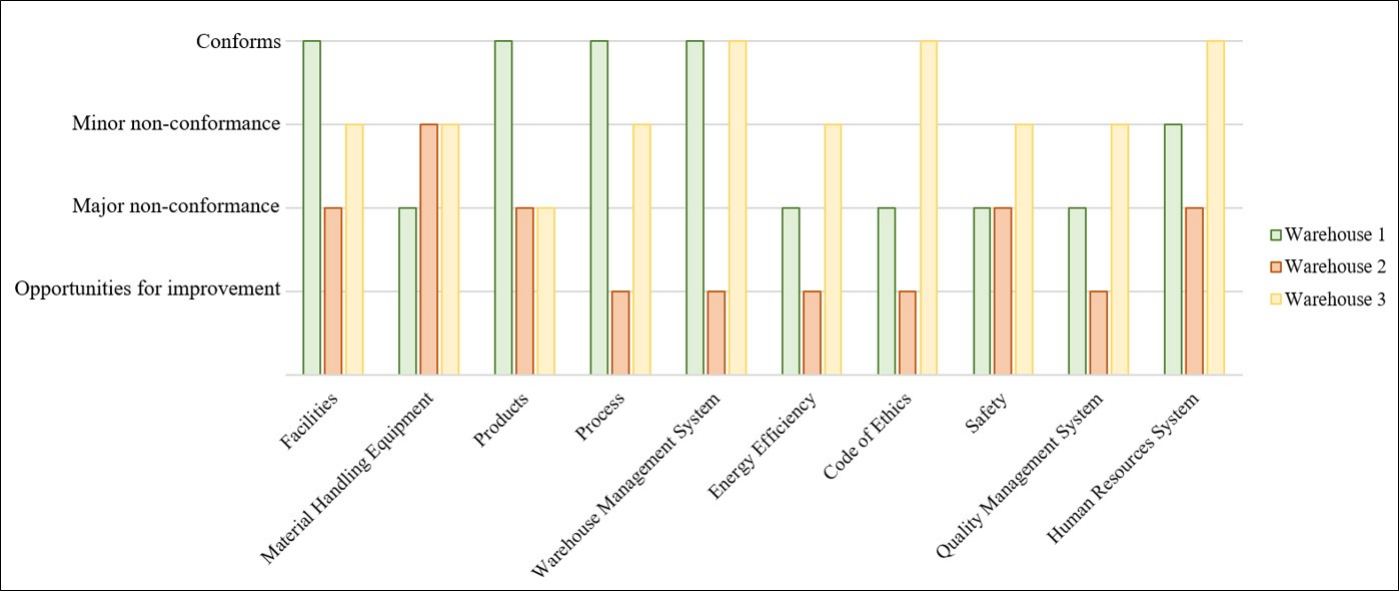
An example of the criteria performance of three warehouses.

To prove the effectiveness of the presented warehouse assessment scheme which was developed using the novel integration of the ANP and the GA, 45 warehouses dealing with different types of products were assessed. Each warehouse was assessed by answering each question defined in the audit checklist by “Conforms”, “Minor non-conformance”, “Major non-conformance” or “Opportunities for improvement”. Fig 7 shows an example of the criteria performance of three warehouses that deal with various types of products. It is worth mentioning that “Warehouse 1”, “Warehouse 2” and “Warehouse 3” deal with pharmacological products, heavy vehicles and insulation plastic equipment, respectively. It is apparent that the performance of the main criteria varies for the warehouses. For instance, the performance values of the “Energy Efficiency” and “Code of Ethics” criteria vary significantly for the three warehouses.

Once the performance value of each criterion was estimated, the overall performance value of each warehouse can be estimated by multiplying the weights estimated in Sub-Section 5.1 for each class by the performance values of the criteria. To illustrate, the overall performance of “Warehouse 1” was determined by using the weights estimated for Class 5 as presented above. The overall performance values for “Warehouse 1”, “Warehouse 2” and “Warehouse 3” are 0.72, 0.28 and 0.76, respectively. It is noticeable that the performance value for “Warehouse 3” was smaller than expected, this being due to the considerable weights given for “Facilities”, “Material Handling Equipment” and “Safety” criteria. In similar manner, the overall performance values for the rest of the warehouses were estimated. It is worth mentioning that such values were in the range of 0.22 to 0.81.

### 5.3 Comparative studies

In order to prove the effectiveness of the proposed warehouse assessment scheme which was developed using the novel integration of the ANP and the GA, five assessment algorithms were utilized in this paper for comparison purposes. Such algorithms are AHP and ANP, as commonly used ones; and interval rough number-weighted aggregated sum product assessment (IRN-WASPAS) [33], criteria importance through inter criteria correlation-weighted aggregated sum product assessment (CRITIC-WASPAS) with interval type-2 fuzzy sets [12] and Complex Proportional Assessment (COPRAS) [34], as newly presented algorithms. In addition to the results obtained by the proposed algorithm that integrates ANP and GA, Table 2 shows the results obtained for three newly warehouses, as examples. Such warehouses perform unsatisfactory, satisfactory and well. Because of the fact the ANP is considered to be the general form of the AHP, it was apparent that the overall performance values estimated by the AHP and the ANP were close. In addition, it was noticeable that overall performance values of “Warehouse 1N” that performs in an unsatisfactory way were overdetermined by IRN-WASPAS, CRITIC-WASPAS and COPRAS. Furthermore, the overall performance values for “Warehouse 3N” were slightly underdetermined by the AHP, ANP, IRN-WASPAS and CRITIC-WASPAS. Likewise, none of the presented algorithms considered was able to numerically represent the strength values of the interrelationships among the criteria and their corresponding sub-criteria. It is worth mentioning at this stage that AHP, ANP and the first stage of the proposed algorithm that integrates the GA and ANP are based on the pairwise comparison which is considered to be computationally expensive in particular when the number of the criteria is relatively large.

**Table 2:** The overall performance values for three warehouses using different algorithms.

Warehouses	Overall Performance					
	AHP	ANP	IRN-WASPAS	CRITIC-WASPAS	COPRAS	Proposed algorithm
Warehouse 1^N^	0.11	0.12	0.27	0.26	0.26	0.13
Warehouse 2^N^	0.61	0.58	0.62	0.64	0.58	0.65
Warehouse 3^N^	0.75	0.77	0.78	0.75	0.80	0.85

*The superscript (N) was used to distinguish these newly warehouses from the warehouses mentioned in Sub-Section 5.2.

## 6. Conclusions

A new assessment scheme for warehouses was developed using the novel integration of the genetic algorithm (GA) and the analytic network process (ANP). The development of such a scheme was performed through various steps, and was validated on 45 warehouses dealing with different product classes. The integration of the GA and ANP allowed users to (i) determine stable relative importance values; (ii) numerically represent the strength values of the interrelationships among the criteria; and (iii) linguistically represent the importance of the criteria and the strength of the interrelationships. Therefore, the warehouse assessment scheme that was based on such a novel integration was successful in identifying those areas that can be considered as competitive advantages and the ones that need to be improved. In addition, the proposed integration can significantly affect various multi-criteria decision-making areas where the optimal course of actions or the best alternative needs to be identified. However, the first stage of the proposed algorithm was based on the pairwise comparison which is considered to be computationally expensive when the number of criteria is relatively large. In addition, there is a strong need to take into account the uncertainties that relate to the subjective information. Likewise, it is worth considering different strength values of interrelationship between two criteria. Therefore, for future work, incorporating dynamic fuzzy logic, instead of the Boolean formalism, within such a framework would be advantageous since fuzzy sets can model vagueness and include uncertainties intrinsically. In addition, a dynamic structure can take into account the dynamic nature of the majority of the multi-criteria decision-making problems.

## Supporting information

S1 Data (XLSX)
